# Combining surface drifters and high resolution global simulations enables the mapping of internal tide surface energy

**DOI:** 10.1038/s41598-025-92662-w

**Published:** 2025-03-28

**Authors:** Zoé Caspar-Cohen, Aurélien Ponte, Noé Lahaye, Edward D. Zaron, Brian K. Arbic, Xiaolong Yu, Sylvie LeGentil, Dimitris Menemenlis

**Affiliations:** 1 Scripps Institution of Oceanography, University of California San Diego, San Diego, CA USA; 2 Univ Brest, CNRS, Ifremer, IRD, Laboratoire d’Océanographie Physique et Spatiale (LOPS), IUEM, 29280 Plouzané, France; 3 Odyssey Team, Inria & IRMAR, Campus Universitaire de Beaulieu, Rennes, France; 4 College of Earth, Ocean, and Atmospheric Sciences, Oregon State University, Corvallis, OR USA; 5 Department of Earth and Environmental Sciences, University of Michigan, Ann Arbor, MI 48109 USA; 6 School of Marine Sciences, Sun Yat-sen University, Zhuhai, China; 7 Jet Propulsion Laboratory, California Institute of Technology, Pasadena, CA USA

**Keywords:** Physical oceanography, Physical oceanography

## Abstract

By dissipating energy and generating mixing, internal tides (ITs) are important for the climatological evolution of the ocean. Our understanding of this class of ocean variability is however hindered by the rarity of observations capable of capturing ITs with global coverage. The data provided by the Global Drifter Program (GDP) offer high temporal resolution and quasi-global coverage, thus bringing promising perspectives. However, due to their inherent drifting nature, these instruments provide a distorted view of the IT signal. By theoretically rationalizing this distortion and leveraging a massive synthetic drifter numerical simulation, we propose a global metric converting semi-diurnal IT energy levels from GDP data to levels comparable to Eulerian datasets (two numerical simulations, and a satellite altimetry IT atlas). We find that the simulation with a dedicated focus on IT representation is the one where the converted Lagrangian levels perform best. This supports renewed efforts in the concurrent numerical modeling of ITs/ocean circulation. The substantial deficit of energy in the IT atlas highlights the inability for altimetric estimates to measure incoherent and fine-scale ITs and strongly supports the need to isolate ITs signature in the data collected by the new wide-swath altimetry mission SWOT.

## Introduction

Internal tides (ITs) are key components of the ocean circulation, as they cause dissipation and mixing, thereby impacting the large-scale ocean circulation^1–4^. Their importance has been recognized for decades and their explicit representation in ocean general circulation models is possible and has been improving for the last decade^5,6^. Moreover, the surface signature of ITs has been flagged as a major issue for the exploitation of emerging satellite sensing—in particular for the Surface Water Ocean Topography (SWOT) mission^7^—as they overlap with the signature of non-wave motions at the submesoscale, while temporal filtering is prevented by the coarse temporal sampling. Understanding internal tide dynamics and quantifying its energetics are therefore of crucial importance and remain insufficient to date.

ITs can propagate over long distances and interact with the turbulent background ocean populated with unsteady jets and eddies. These interactions alter IT propagation and result in a loss of coherence (reduction of phase-locking with the generating source—the barotropic tide). Part of the IT signal will then be incoherent (non phase-locked), characterized by an incoherent IT variance and an incoherent time scale. As a consequence, the IT estimates based on sea level measurements from satellite altimetry, which rely on multi-years long time series to dealias IT high-frequency signals, are limited to their coherent contribution and missing part of the IT signal^8,9^. Estimates of total (coherent and incoherent) semi-diurnal tide variance have been obtained from along-track altimetry data (for mode-1 IT)^10^, and using Argo float measurements^11^, moorings^12^ and cruises^13^. Estimates of the incoherent tide variance, based on along-track altimetry data and Argo floats, were found to reach from 44 to 85% of the total energy^10,11^. These estimates are however limited to specific areas due to the restrained spatial coverage.

Numerical models have recently become able to explicitly represent internal tide fields in high resolution realistic simulations of order a year long and at basin or global scale^14^. The comparison of numerical simulations to other datasets, notably moorings^12^ or altimetry^15^, supports their potential to simulate both coherent and incoherent internal tides. While these simulations provide valuable insights on the IT dynamics, it has been shown that numerical aspects such as parameterized wave drag can have a strong impact on the simulated IT field^16^. To validate these numerical models, and complement satellite altimetry, continuing effort to find appropriate data is called for in order to identify the total global IT kinetic energy, i.e., including both coherent and incoherent internal tides for all vertical modes.

In that regard, the quantification of the IT field through globally deployed surface drifters from the Global Drifter Program, GDP^17^, is particularly relevant. Indeed, it provides hourly data on drifters’ positions, from which currents can be estimated across a wide range of time scales (including motions at the typical semi-diurnal tide period of $\sim 12$ h) at global scale. Drifters have a drogue at 15 m depth that ensures that they follow the ocean surface currents and not the surface winds. Estimates of kinetic energy in the tidal frequency bands are thus possible from this dataset^18,19^. However it has been identified that the Lagrangian—i.e. along-flow—perspective can bring some distortion with respect to the Eulerian—i.e. fixed-point—one^20,21^. This distortion is caused by the advection of the drifters by the low-frequency flow, with respect to the horizontal wavelength of the internal tides. Drifters can be advected by the mesoscale currents in such a way that the distance they travel over a time period equal to the ITs decorrelation timescale is several times larger than the ITs wavelength. This distortion, coined ”apparent incoherence”, must therefore be addressed if one seeks a reliable estimate of the internal tide energy to compare to Eulerian estimates.

We show here how theoretical model and realistic simulation may be used to understand and predict the differences between Eulerian and Lagrangian data. We propose a method to estimate semi-diurnal internal tides kinetic energy from the GDP hourly dataset and convert those levels so they can be compared to Eulerian diagnostics. Using a state-of-the-art high-resolution numerical simulation of the world ocean, the Massachusetts Institute of Technology general circulation model (MITgcm) LLC4320, populated with surface Lagrangian particles, we first construct model-based maps of IT surface kinetic energy and identify the relationship between Lagrangian and Eulerian diagnostics. We then propose and validate a simple global metric accounting for the Lagrangian distortion, that allows us to convert Lagrangian-based kinetic energy estimates via a conversion factor. These converted estimates are then compared with estimates from numerical models (MITgcm and the Hybrid Coordinate Ocean Model—HYCOM) and from altimetry data (High Resolution Empirical Tide—HRET).

## Results

### Quantifying and explaining Lagrangian biases with high resolution simulation and theory

**Fig. 1:**
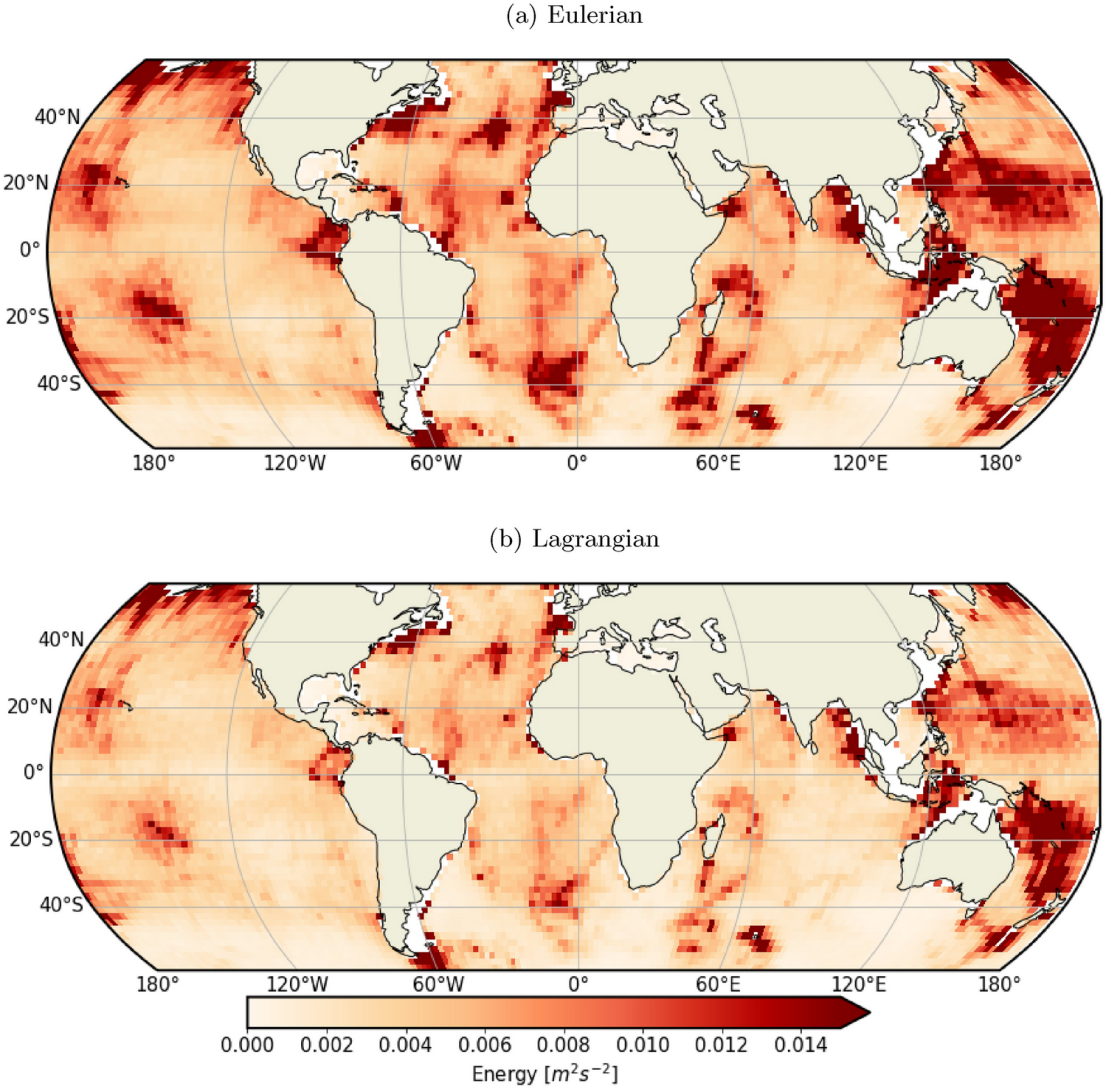
Maps of (**a**) Eulerian and (**b**) Lagrangian kinetic energy levels in the semi-diurnal band computed from LLC4320 surface outputs and simulated drifter trajectories from November 15th, 2011 to November 9th, 2012. The energy levels are averaged over entire time series length and over $2^{\circ } \times 2^{\circ }$ spatial bins, following 1 and 2.

**Fig. 2:**
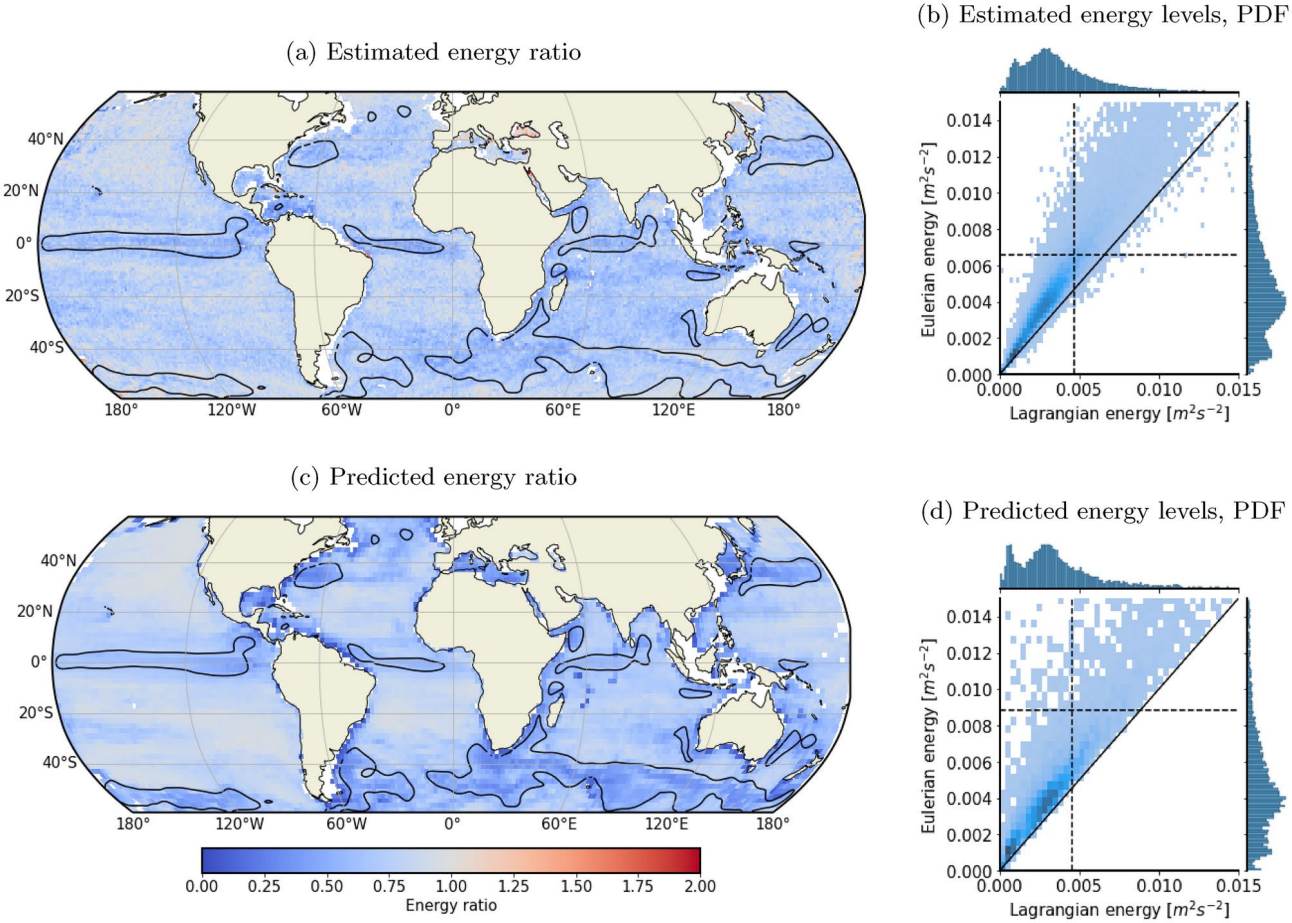
Comparison between estimated Eulerian surface kinetic energy (from LLC4320) and estimated/predicted Lagrangian surface kinetic energy averaged over time (from November 15th, 2011 to November 9th, 2012) and $1^{\circ } \times 1^{\circ }$ horizontal bins. Maps of (**a**) estimated and (**c**) predicted Lagrangian to Eulerian energy ratio in the semi-diurnal band computed from LLC4320 surface outputs and simulated drifter trajectories. Black contours define regions in which the low frequency kinetic energy is larger than $0.1\,\hbox {m}^{2}\,\hbox {s}^{-2}$. Joint plots of the distribution of (**b**) estimated and (**d**) predicted (x-axis) Lagrangian and (y-axis) Eulerian energy levels are also plotted in the right panels. Dashed black lines represent mean Eulerian and Lagrangian energy values.

The state-of-the-art global tide resolving numerical simulation LLC4320 (based on the MITgcm model^22^;) and a synthetic drifter release based on LLC4320 velocity outputs are leveraged to produce a unique comparison between Eulerian (fixed-point) and Lagrangian (drifter/along-flow) semi-diurnal internal tide kinetic energy (Fig. 1, Section “Methods”). As, expected, both Lagrangian (Fig. 1a) and Eulerian (Fig. 1b) energy levels exhibit maxima at internal tide generation hotspots, near oceanic ridges and islands (e.g., mid-ocean ridges, South China Sea, etc.) with values up to $\sim 0.015\,\hbox {m}^{2}\,\hbox {s}^{-2}$. A global reduction of drifter energy levels is observed compared to Eulerian ones, with an average of approximately 75$\%$ of the Eulerian energy recovered in the Lagrangian framework (Figs. 1 and 2a and b). This could impact our ability to compare drifters observation and Eulerian-based estimates and needs to be explained and accounted for.

**Fig. 3:**
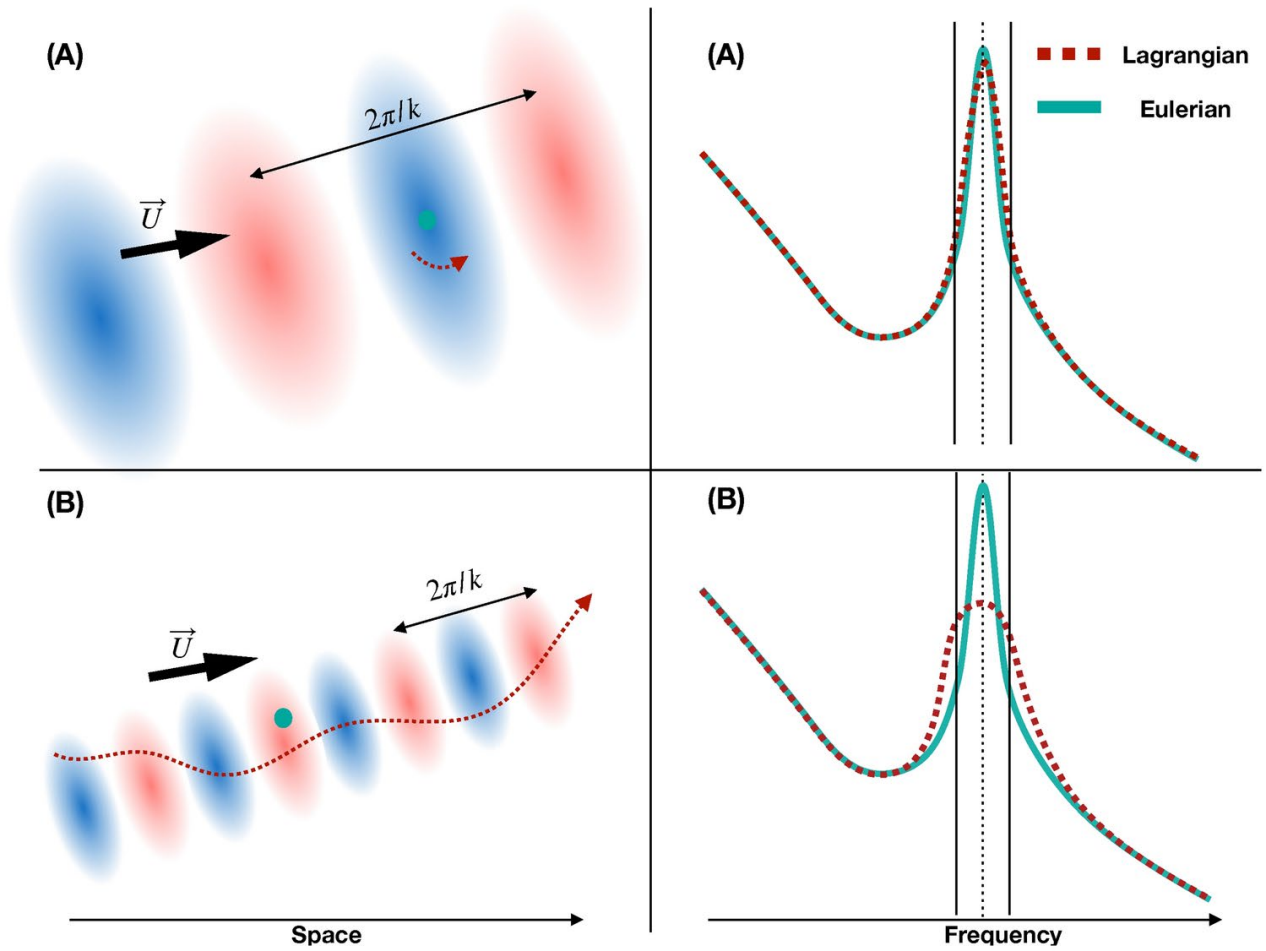
Schematic description of the bias introduced by drifter displacements and how this bias impacts the energy levels found in a fixed frequency band. Two examples are shown, labeled (**A**) and (**B**). (**A**) corresponds to weak and (**B**) to strong drifter advection by the low-frequency background flow. Left panels represent the waves signature and the drifter displacement, represented by the dashed red curve, compared to the wavelength $2\pi /k$. $\vec {U}$ represents the background low-frequency flow. Right panels represent the same case scenario in the frequency domain with schematic power spectra around a central frequency (represented by the dotted line). Solid vertical black lines correspond to the limits of a fixed frequency band.

The Lagrangian to Eulerian energy ratio is referred to as “estimated energy ratio” in the rest of the study. This ratio varies geographically, ranging from about 0.5 to unity. It is clearly modulated by the intensity of low frequency motions with lowest energy ratio observed in low frequency energetic areas, e.g. Equatorial currents, Gulf Stream, Kuroshio (Fig. 2a). This dependence of the bias between Lagrangian and Eulerian energy levels on the low-frequency flow magnitude is explained by Caspar-Cohen et al.^21^. It is caused by the combination of two effects: (1) the distortion of ITs temporal signature induced by drifters’ motions relative to ITs horizontal structure , (2) the filtering of the velocity signal in a fixed frequency band. The former effect is related to the displacement-induced projection of spatial variability into the temporal one, which is a well-known and more general feature associated with Lagrangian observations^23^. The magnitude of this distortion thus depends on the distance traveled by a drifter over an IT time period relative to the IT horizontal wavelength, and this distance directly depends on the mean flow strength (Fig. 3 left panels). Caspar-Cohen et al.^21^ showed that the distortion leads to more rapid modulations of ITs which, in the frequency domain, translates into wider peaks in the Lagrangian spectra (Fig. 3, right panels and Fig. S2 in Supplementary information). Once integrated across the IT frequency band, Lagrangian kinetic energy estimates will thus tend to be weaker than Eulerian estimates. In the case of small drifter displacements (labeled (A) in Fig. 3), the drifter behaves as an Eulerian observer (e.g. a mooring) and measures purely temporal fluctuations. Lagrangian and Eulerian spectra match (Fig. 3, label A, right panel), resulting in similar band-integrated energy levels. Conversely, in the case of large drifter displacements (labeled (B) in Figure 3), the wave spatial variability is projected into the temporal one, resulting in apparent incoherence with wider Lagrangian spectral peaks compared to Eulerian ones (Fig. 3, label B, right panel), and ultimately lower Lagrangian energy estimates. This effect is mainly limited to 75$\%$ of the Eulerian energy, but can be stronger in regions of strong currents (such as some of the ones previously listed).

The apparent incoherence theoretical model of Caspar-Cohen et al.^21^ is further exploited to predict the Lagrangian to Eulerian kinetic energy ratio—referred to as “predicted energy ratio” (Fig. 2c and d). Its prediction is further described in “Methods” Section. Estimated and predicted energy ratios compare well visually with predictions of energy ratio minima in terms of their values ($\sim 0.5$) and locations. The differences observed are expected as the theoretical model was developed to explain apparent incoherence as a kinematic process and uses some simplifications, notably averaged motions properties (wavenumbers, decorrelation timescales, etc)^21^. Despite this, the spatial structures of the predicted and estimated energy ratios match in approximately 93% of instances. This strongly supports the fact that the smaller Lagrangian-based estimates are indeed linked to apparent incoherence and purely caused by the entanglement of spatial and temporal variability in Lagrangian-based estimates and the associated widening of Lagrangian spectra. A conversion from Lagrangian to Eulerian framework is thus necessary in order to compare them to Eulerian-based estimates.

### Applications: comparing Eulerian datasets to ground truth energy levels

Now that the differences between Eulerian and Lagrangian-based energy estimates have been rationalized and predicted, we introduce a conversion factor to obtain reference energy levels from global, high temporal resolution in situ drifter dataset. In accordance with theory, we assume that the Lagrangian to Eulerian energy ratio depends on the strength of the low-frequency flow (defined as motion with timescales larger than 2 days) and internal tide spatial scales, both of which we assume are correctly predicted by the LLC4320 simulation (see Yu et al.^18^ for a quantitative description). This ratio can then be used as a conversion factor for Lagrangian observations. From there, converted Eulerian-like energy estimates (Fig. S1a in Supplementary information) are obtained by multiplying Lagrangian-based energy levels with a conversion factor defined as the estimated energy ratio in LLC4320 (Fig. 2a).

**Fig. 4:**
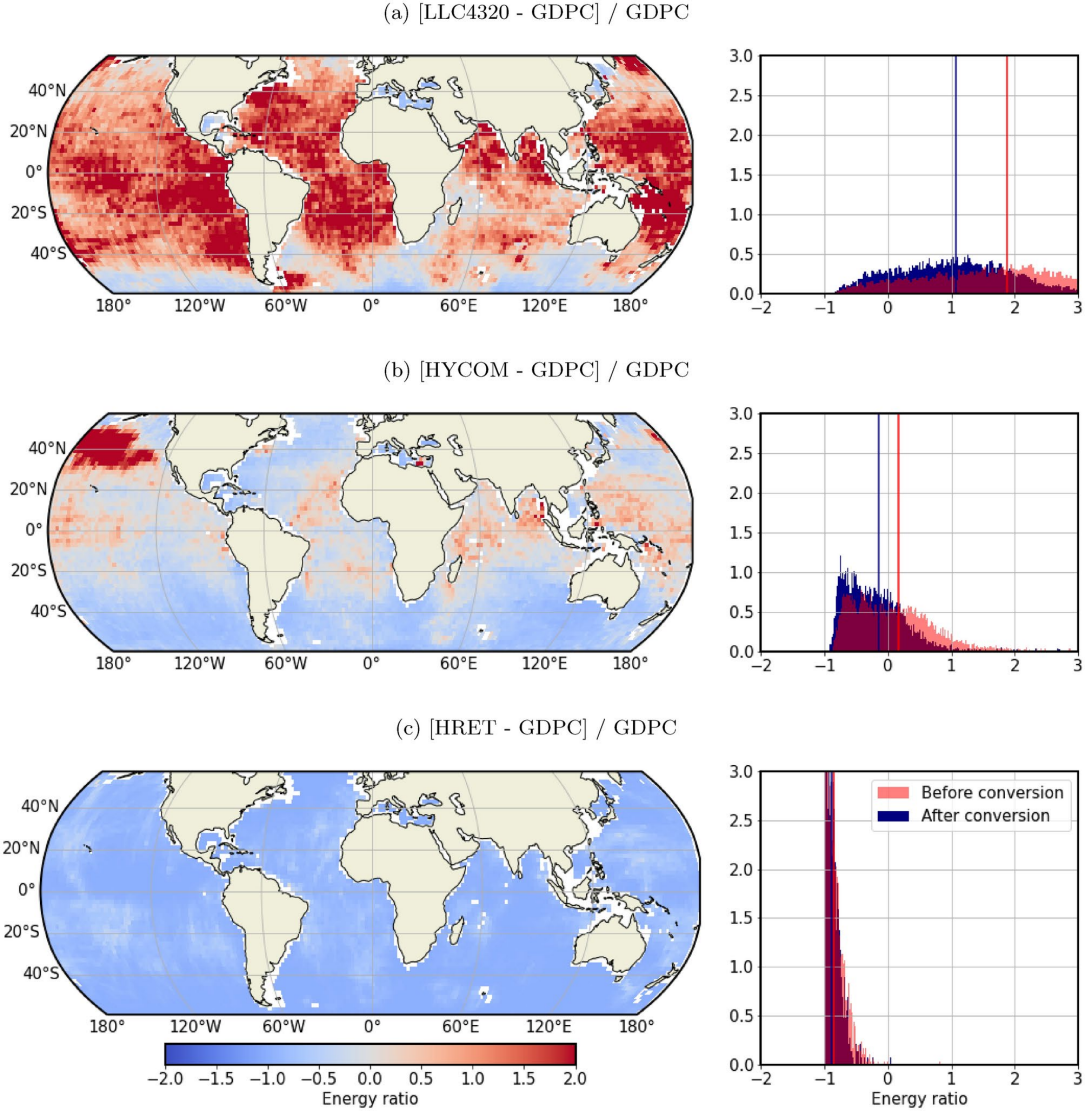
Comparison of semi-diurnal kinetic energy estimated from GDP dataset to the ones from LLC4320, HYCOM and HRET. (Left panels) Maps of the surface semi-diurnal kinetic energy differences between (**a**) LLC4320, (**b**) HYCOM and (**c**) HRET and the converted energy levels from GDP surface drifters normalized by converted GDP energy levels. (right panels) The distributions of the difference between each dataset and (blue) converted and (red) biased energy levels from GDP data are shown. Mean energy differences are represented by the colored vertical lines.

This conversion is thus applied to the energy estimate from the in situ Lagrangian observations of hourly surface velocity provided by the GDP (Elipot et al.^17,24^, Section “Methods”). The converted energy levels (GDPC) may be used as reference energy levels and compared to Eulerian-based estimates giving us a unique tool to directly validate and complement estimates from numerical models and along-track altimetry. We next use energy levels estimated in the same frequency band in two high resolution global numerical simulations, LLC4320 (Fig. 4a) and HYCOM (Arbic^14^; Arbic et al.^19^; Figs. 4b and  S1b in Supplementary information, and Section “Methods”). In addition, we compare our dataset to estimates from altimetry (Zaron et al.^8^; Fig. 4c).

**Fig. 5:**
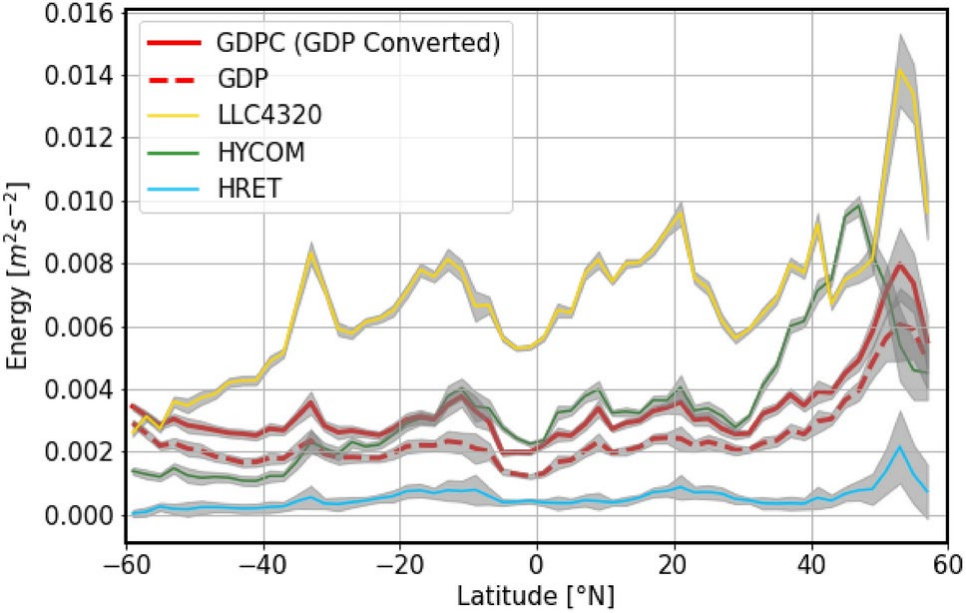
Zonal average of the surface semi-diurnal kinetic energy estimated from LLC4320 (yellow), HYCOM (green), GDP (dashed red), converted GDP (solid red) and HRET (blue). Grey shading correspond to error due to spatial sampling (i.e. standard deviation).

Despite similar input data types and processing, both numerical simulations exhibit significant differences when compared to semi-diurnal converted energies. LLC4320 energy levels overall overestimate GDP converted energy levels by a factor 2 on average (Fig. 4a). Both LLC4320 and converted GDP energy levels follow a similar dependence on latitude (Fig. 5 yellow and red curves), supporting the hypothesis of general phenomena causing this overestimation. Arbic et al.^19^ attributed this issue to the lack of a parameterized topographic internal wave drag in LLC4320 which has been shown to be necessary for accurate tides in HYCOM^5,14,16,25^. Comparing simulation outputs to converted energy levels instead of the biased (e.g., Lagrangian) ones attenuates this overestimation, decreasing from a factor 3 of the original dataset to a factor 2 of the converted one (Fig. 4a, right panel). In comparison, the HYCOM simulation shows a better match with converted energy levels, representing 87$\%$ of converted levels. Differences between HYCOM and converted GDP energies highlight regional differences with, for instance, an underestimation below $40^{\circ }$S where HYCOM energy levels represents 37$\%$ of the GDP energy, and an overestimation in the North eastern Pacific area (energy levels five times higher than the converted GDP energy). Arbic et al.^19^ attributed the latter anomaly to numerical instabilities. The area between $-30^{\circ }$ and $30^{\circ }$N shows a particularly good concordance with converted levels, visible especially in the zonal average (Fig. 5 green and red curves), with an average overestimation of the converted GDP energy by a factor 1.03, i.e., only $3\%$ difference compared to GDPC energies. The comparison of our converted dataset to this simulation highlights again the importance of the conversion process as energy levels would have been overall overestimated in HYCOM if compared directly to GDP energy levels, 87$\%$ of converted levels versus 117$\%$ of the original GDP dataset.

Reference energy levels also open the door to the quantification of IT incoherent energy fractions. Due to their low temporal resolution, IT atlas derived from satellite altimetry are indeed limited to coherent IT and few vertical modes. The incoherent energy has been estimated previously to account for a significant fraction of the total tidal energy 44^10^–$68\%$^15^. This remains true even in the case of advanced products such as High Resolution Empirical Tide (HRET)^8^. Indeed, while HRET successfully represents IT generation hotspots and main area of interest (Fig. S1c in Supplementary information), its energy levels strongly depend on its ability to include incoherent and high modes tides in this representation. In the case of mode-1 internal tides and considering only the two main components, $M_2$ and $S_2$, kinetic energy from HRET represents only 11$\%$ of the reference energy levels (Figs. 4c and 5 blue curve). As further discussed in “Discussion and Methods” Sections , the fundamental difference of data processing between HRET and GDP dataset explains this large difference. This result highlights the significance of including incoherent and/or contributions of higher modes as well as the necessity to use in situ observations to complement satellite altimetry.

## Discussion

Our study confirms the relevance of apparent incoherence for Lagrangian based mapping of the semi-diurnal internal tide. Its existence was speculated by Zaron and Elipot^20^ and Arbic et al.^19^ and verified in idealized simulations and modeled theoretically by Caspar-Cohen et al.^21^. Apparent incoherence and the associated spectral widening of the semi-diurnal peak resulting from drifter displacements is found to lead, without adequate treatment, to an average low bias of 25% of semi-diurnal Lagrangian-based estimated when compared to Eulerian-based ones. This difference is the largest in areas of energetic low frequency variability, where Lagrangian energy levels represents only 60$\%$ of the Eulerian energy levels, in accordance with theoretical predictions.

Motivated by this successful theoretical description, we proposed a conversion of the GDP based estimates of semi-diurnal energy distribution to an Eulerian-like distribution. This method relies on the comparison between Lagrangian and Eulerian semi-diurnal energy levels in a state of the art numerical simulation of the ocean circulation. Our approach essentially remains robust to an overall bias in semi-diurnal variability energy for estimates averaged over hundreds of kilometres ($1^{\circ }$ horizontal resolution at best) and long time series (at least a year). We critically assume that the numerical simulation is able to reproduce the internal distribution across spatial scales.

The latter assumption could be partly tested in latter studies at isolated spots with mooring data^12,26–28^, or altimetric observations via coherent internal tides^29–32^. Future studies focusing on improving the current limitations may be developed from other numerical global simulations, such as HYCOM, and regional in situ observations and simulations. Efforts have very recently been made to simulate drifters in HYCOM, which should make a possible comparison of both datasets in the future^33^. In the present study, we however consider that the use of a conversion factor based on LL4320 allows an initial understanding of the areas of significant energy underestimation in the Lagrangian dataset before conversion. LLC4320 is indeed a realistic simulation with high resolution, resolving the main elements responsible for drifters advection, and subsequent apparent incoherence found in the data, as described by (4). Statistical estimates over long time series (Fig. 5, solid red curve) allow a better comparison to both HYCOM and LLC4320 compared to the estimates before conversion (Fig. 5, dashed red curve). This limits the study to a statistical focus, without considering seasonal or shorter time period and local spatial variability (scales smaller than $2^{\circ }$). The effect of short lived strong winds or surface currents would require dedicated focus using regional datasets and simulations and are beyond the scope of the present study. In particular seasonal variability, notably of winds and mesoscale, may impact significantly the drifters motions and the way they capture internal tides signal. A dedicated study and the use of regional simulations would be necessary to define an adequate conversion factor. Other metrics may also be developed to answer different needs of future studies, i.e. better spatial resolution, inclusion of extreme weather, etc.

Our original approach enabled us to reassess the accuracy of the semi-diurnal internal tide variability predicted by two global tide-resolving numerical predictions (MITgcm LLC4320 and HYCOM). Previous results are confirmed: MITgcm LLC4320 semi-diurnal energy is higher than in situ observations by a factor of about two on average (equivalent to $8\times 10^{-3}\,\hbox {m}^{2}\,\hbox {s}^{-2}$). HYCOM exhibits relatively little bias on average but regional modulations emerge: underestimated energy outside the tropics, overestimation in the tropics, and anomalous energy excess in the North Pacific caused by numerical instability. Reference energy levels Eulerian-like estimates using a conversion of Lagrangian observations highlight models and areas for which configurations are seemingly best suited to successfully describe IT dynamics. In addition, our study emphasizes the need to carefully factor potential biases when using in situ observations to validate these simulations. This assessment of the accuracy of numerical simulations, in regard to IT kinetic energy, is potentially limited by the different temporal coverage between numerical simulations and GDP ($\sim$ one year against several decades). We argue that at least a year is covered by numerical simulations which mitigates seasonal fluctuations of the semi-diurnal IT energy^34^. Inter-annual fluctuations however should be investigated in the future.

A second direct application of our estimate of semi-diurnal internal tide energy is to provide new estimates of the IT *incoherent* kinetic energy via comparisons with altimetry-based estimates of the coherent energy (HRET). The averaged incoherent, and modes higher than 1, energy is about $5\times 10^{-4}\,\hbox {m}^{2}\,\hbox {s}^{-2}$, which is 89% of the total energy on average. This metric may be leveraged to assess the ability of tidal models to reproduce coherent and incoherent internal tides independently, and constitute a useful background value for internal tide data assimilation efforts^35–37^.

Two points that may be investigated in the future are the presence of non-tidal motions in the semi-diurnal frequency band, e.g., submesoscale low-frequency motions, and, on the contrary, the risk to exclude some of the tidal energy. Two parameters control this: the form of the spectra of low-frequency motions, mostly determined by the decorrelation timescale, and the width of the frequency band chosen for the data processing. An investigation showed that a theoretical spectral form for low-frequency motions and tests of different frequency bands could provide a satisfying frequency bandwidth (Fig. S3). This results in a generic choice that successfully reduced the fraction of low-frequency energy present in the semi-diurnal band and its impact on the comparison of Lagrangian datasets with Eulerian-based estimates. This choice could be more finely defined in local studies, where a more precise optimal bandwidth could be necessary. However, this fraction of energy and/or other source of contamination will change depending on the frequency of interest of a study and optimum bandwidth should be carefully defined for each study case.

Overall, this study highlights a recent surge of efforts and substantial results around the mapping of internal tides in the ocean in general and from drifter data in particular. Leads for future studies are numerous ranging from the application of present methods to diurnal tides (although apparent incoherence is expected to be weaker, as associated length scales are larger, and part of the signal may be non-tidal; Arbic et al.^19^), to per-vertical mode description of the effect of apparent incoherence. SWOT, its fast sampling phase in particular, may provide unprecedented details of internal tide dynamics which combined with drifter data could lead to unprecedented descriptions of the ocean internal tide variability. This could have a long-standing impact on our ability to represent internal tides explicitly in high resolution numerical simulations or implicitly via the parametrization of their effects in climate numerical simulations^38–41^.

## Methods

### LLC4320 and GDP data processing

LLC4320 is a global-ocean configuration based on the MIT general circulation model (MITgcm)^22^. Its grid resolution is $1/48^{\circ }$ horizontally, i.e., around 2 km (1 km in the Arctic, 2.4 km at the Equator) with 90 vertical levels. Outputs have been produced with hourly resolution. Surface boundary conditions are based on $0.14^{\circ }$ European Centre for Medium-Range Weather Forecasts (ECMWF) atmospheric operational model analysis 6 hourly product, starting in 2011. Tides are forced with 16 major constituents. An erroneous $10\%$ overestimation of the tidal forcing has been reported in Arbic et al.^19^. LLC4320 dataset is approximately one year long, beginning on the 15th of November 2011 and ending on the 9th of November 2012. Drifter trajectories are computed offline from the hourly surface velocity fields using Ocean Parcels^42^. A 4th order Runge-Kutta temporal integration scheme and the spatial interpolation scheme TRACMASS are used^43^. Normalized amplitude and phase errors associated with the 4th order Runge-Kutta temporal integration are about $(\omega dt)^6/144\sim 0.01\%$ and $(\omega dt)^4/120\sim 0.01\%$ for semi-diurnal periodic signals^44^. Drifters are initially deployed every 50 grid points ($\sim 1^{\circ }$) in latitude and longitude. Every 10 days, drifters are released at initial drifter locations if the closest drifter exceeds the initial closest neighbour separation. The number of drifters in the simulation hence increases from about 60 000 to 100 000 at the end of year-long simulation. This reseeding strategy enables to obtain above 8000 drifter positions per $1^{\circ } \times 1^{\circ }$ spatial bin over the time series length outside of Equatorial area and above 5000 drifters at the Equator. The resulting Lagrangian horizontal velocities were compared to the Eulerian velocities interpolated on the drifters trajectory in areas of low apparent incoherence, i.e. in cases where we expect no differences. Both fields were similar, which supports the validity of the simulated drifters.

The in situ drifter dataset used in “Results” Section are the surface velocity fields provided by the GDP with an hourly time resolution. In our study data from both Argos and GPS-tracked drifters were used^17^. The error on the entire velocity dataset is estimated between 2 and 5 cm $\hbox {s}^{-1}$ over the total velocity^18^. Assuming the error is proportional to the amount of energy in a given frequency band, i.e. the error is higher when velocity is higher, its effect on the energy levels will remain lower than the bias introduced by the change of Lagrangian to Eulerian perspective.

The same data processing is applied to both simulated and in situ datasets in order to extract semi-diurnal variability from velocity time series. The first step is to bandpass filter the raw signal in the semi-diurnal frequency band defined by its central frequency $\omega _c=(\omega _{M_2}+\omega _{S_2})/2 \simeq 1.97$ cpd and bandwidth $\Delta \omega =0.4$ cpd. The Hilbert transform is then applied to filtered time series. The resulting analytical signal is multiplied by $\exp {(-i\omega _c t)}$ leading to the demodulated tidal signal ${\tilde{u}}(t)$. As illustrated in Arbic et al.^19^, energy estimates may be sensitive to the choice of bandwidth. The present choice is motivated by synthetic experiments and results from a trade-off aiming at reducing the imprint of background energy while including the majority of the semi-diurnal tidal signal.

Averaged kinetic energy in the semidiurnal band are then obtained from the demodulated horizontal velocity time series, ${\tilde{u}}$ and ${\tilde{v}}$ for the zonal and meridional velocity respectively. For Eulerian time series, this averaged energy is given by:

1 $$\begin{aligned} KE_{E,high} = \frac{1}{2}<\overline{\tilde{u}_E^2+\tilde{v}_E^2}>_b \end{aligned}$$

where $<.>_b$ is the horizontal bin average and $\overline{. }$ is the time average. The denotation “high” in this case refers to the kinetic energy in the frequency band used for the filtering, $\omega _c\pm \Delta \omega$. For Lagrangian model and in situ time series, the energy is computed according to:

2 $$\begin{aligned} KE_{L,high} = \frac{1}{2}<{\tilde{u}_L^2+\tilde{v}_L^2}>_{b,t} \end{aligned}$$

where $<.>_{b,t}$ is the time and horizontal bin average.

### HYCOM data processing

The dataset from the HYCOM simulation was processed outside of the scope of this study by Arbic et al.^19^ who give a complete description of both data and method. The HYCOM simulation has a $1/25^{\circ }$ horizontal resolution with 41 vertical levels. The tidal forcing accounts for the 5 largest tidal components, including the three main semi-diurnal components, M2, S2, and N2. Outputs are provided with hourly time resolution for about one year starting on 1 January 2014.

Kinetic energy is estimated from frequency rotary spectra which are computed by splitting the complex velocity time series $u + iv$, where *u* and *v* denote zonal and meridional velocities respectively, into 60-day windows overlapping by 50%^19^. For each temporal window, time series are detrended and multiplied by a normalized Hann window. Individual discrete Fourier transform are then computed and multiplied by their complex conjugates. Averages over all windows and within $1^{\circ } \times 1^{\circ }$ bins lead to time-averaged kinetic energy spectra. These spectra are then integrated in the 1.8–2.2 frequency band, thereby providing the maps of kinetic energy presented in “Results” Section.

The data processing differs from the one applied to LLC4320 and GDP datasets. In order to investigate the potential impact of this difference on the comparison between GDP energies and HYCOM ones, both data processing methods were compared with LLC4320 data. Observed differences are mostly noise-like and lower than differences reported in “Results” Section (2$\%$ due to the noise in average against 6$\%$ caused by the difference of dataset).

### High resolution empirical tide (HRET)

HRET processing is described in Zaron^45^ and Zaron et al.^8^. HRET is an internal tide atlas based on satellite altimetric data mapping the sea surface height (SSH) associated with internal tides component^8,45^. Estimation of variance are obtained for the $M_2$ coherent IT signal. To this signal we added an estimation of the variance of the $S_2$ coherent IT using theoretical equilibrium tides amplitudes.

3 $$\begin{aligned} KE = KE_{M_2}(1+\frac{a_{S_2}}{a_{M_2}}) \end{aligned}$$

where $KE_{M_2}$ is the energy estimation from HRET, $a_{S_2}$ is the equilibrium amplitude of $S_2$ and $a_{M_2}$ is the equilibrium amplitude of $M_2$.

The estimation of energy based on HRET accounts for the coherent signal of two tidal components, $M_2$ and $S_2$. HRET thus provides a fraction of the signal that can be obtained via integrated spectra or bandpass filtering, and is therefore expected to provide lower energy levels compared to data sources that account for the full tidal signal (e.g. LLC4320, HYCOM, GDP). The comparison between HRET diagnostics and other data sources highlights the fraction of internal tide energy not represented in satellite climatologies.

### Predicting apparent incoherence

In “Results” Section, we present a prediction of the Lagrangian to Eulerian energy ratio. This prediction is based on the study developed in Caspar-Cohen et al.^21^ which provides a theoretical model for the Lagrangian autocorrelation, following:

4 $$\begin{aligned} {\tilde{C}}_L(\tau ) = {\tilde{C}}_E(\tau ) e^{-k^2\sigma ^2(\tau )} \end{aligned}$$

where ${\tilde{C}}(\tau )$ is the Eulerian autocovariance, k the internal tide horizontal wavenumber and $\sigma$ a prediction of drifters’ displacement depending on the low frequency motion amplitude and decorrelation timescale. Internal tides and low frequency motion properties (energy and decorrelation timescales) are estimated from the Eulerian outputs of LLC4320 simulation, following the fitting method described in Caspar-Cohen et al.^21^. From these Eulerian estimates, Eulerian autocovariance is computed, and the Lagrangian autocovariance predicted. Eulerian and Lagrangian spectra, noted ${\tilde{E}}_e$ and ${\tilde{E}}_l$ respectively, are then estimated via their relationship with autocovariance functions:

5 $$\begin{aligned} E(\omega ) = \int _{-\infty }^\infty C(\tau )cos(\omega \tau )d\tau \end{aligned}$$

where C is an autocovariance function. Estimates of the Eulerian and Lagrangian energy fields can then be inferred from these spectra by integration in a fixed bandwidth.

6 $$\begin{aligned} KE_{E,high}&= \int _{\omega _c-\Delta \omega /2}^{\omega _c+\Delta \omega /2} {\tilde{E}}_{e}(\omega )d\omega , \end{aligned}$$

7 $$\begin{aligned} KE_{L,high,predicted}&= \int _{\omega _c-\Delta \omega /2}^{\omega _c+\Delta \omega /2} {\tilde{E}}_{l}(\omega )d\omega \end{aligned}$$

where $\omega _c$ is the central frequency of the filter and $\Delta \omega$ its bandwidth. Consequently the energy ratio referred to as “predicted energy ratio” in “Results” Section corresponds to $KE_{L,high,predicted}/KE_{E,high}$ (Fig. 2a and b) and is compared to the “estimated energy ratio”, $KE_{L,high}/KE_{E,high}$ (Fig. 2c and d). (7) estimated from (4) links the low-frequency flow properties to kinetic energy estimates in Lagrangian data and highlights it as the main driver of the differences between Eulerian and Lagrangian estimates of kinetic energy. As developed in “Results” Section, both ratio are similar, presenting the same locations of minimal values, i.e. high apparent incoherence effect. Some differences are visible especially in strongly turbulent currents, such as the Antarctic Circumpolar Current and the Kuroshio. In these currents, the predicted ratio is lower than the estimated one. This is explained by the presence of balanced mesoscale energy at high frequency in the case of strong low-frequency currents. The amount of energy contaminating the semi-diurnal band is not taken into account in our model which explains the underestimation observed. This is the main reason for which the estimated energy ratio is the one used as the conversion factor in the second part of “Results” Section. The model also presents other limitations as it is developed for statistical purpose and does not withstand in studies focusing on local phenomena. Using this theory locally would require further theoretical work, including shorter timescales elements, such as strong winds, small scale low-frequency currents.

## Supplementary Information

Supplementary Information.

## Data Availability

The IT energy levels estimated from LLC4320 and GDP datasets are provided at, https://doi.org/10.5281/zenodo.10851200. The HRET tide model is available to reviewers at the URL, https://ingria.ceoas.oregonstate.edu/fossil/SMCE/dir?ci=tip. A manuscript describing the model is under review at JTech. A DOI for the model will be provided if this manuscript is accepted. The Matlab code used to process HYCOM outputs and the results used in this paper are provided in Arbic et al.^19^; https://doi.org/10.7302/PTG7-YW20.
