# Supplementary material for: Combining surface drifters and high resolution global simulations enables the mapping of internal tide surface energy
Source: Sci Rep. 2025 Mar 28;15:10672. doi: 10.1038/s41598-025-92662-w (PMC11950310; doi:10.1038/s41598-025-92662-w)
Supplement: Supplementary file 1 — Supplementary Information. [file 41598_2025_92662_MOESM1_ESM.pdf]

Supplementary information

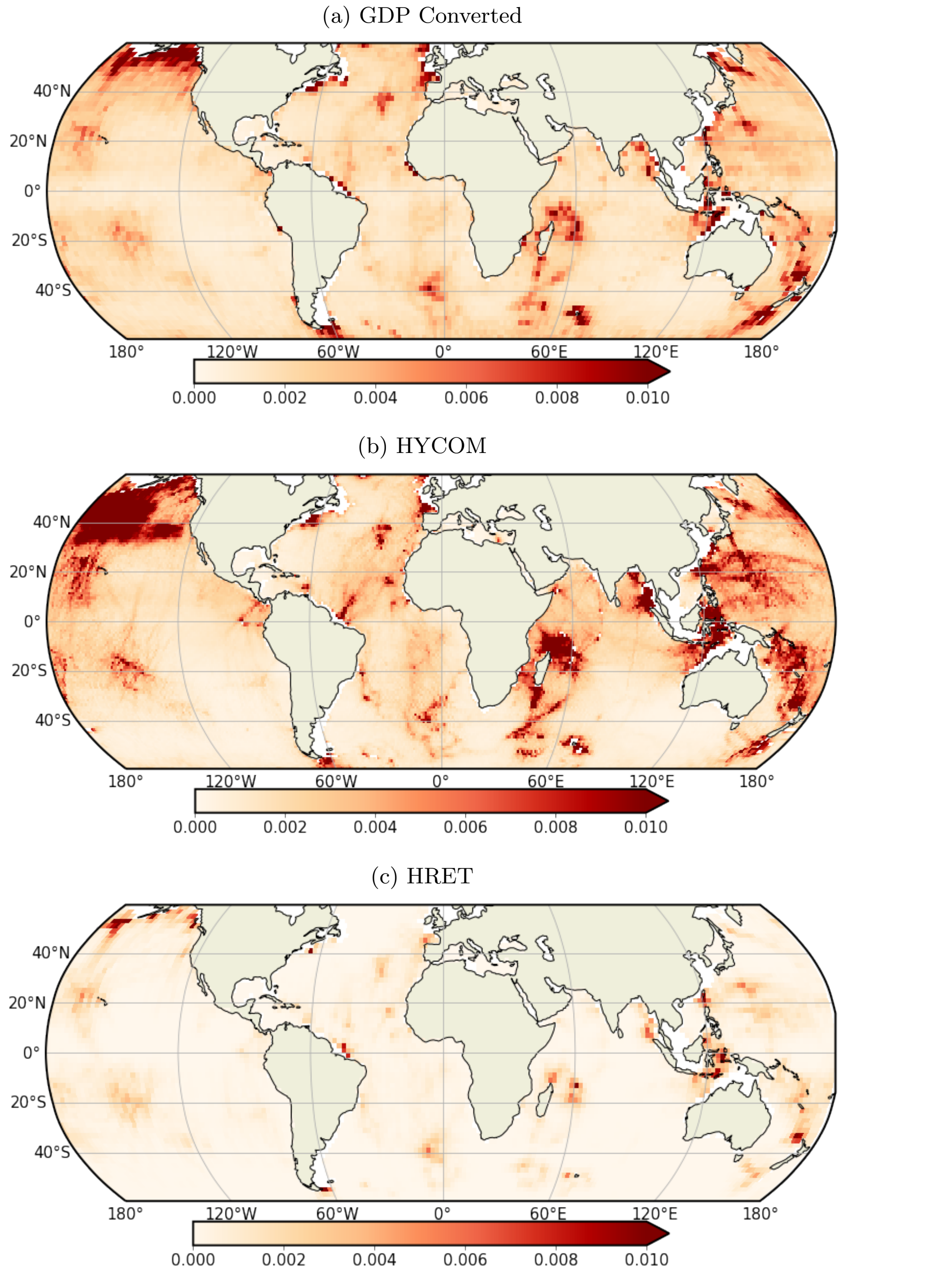

S 1: Maps of semidiurnal IT kinetic energy estimated from (a) GDP, (b) HYCOM and (c) HRET datasets. The energy levels are averaged over time and over 2°x2° spatial bins.

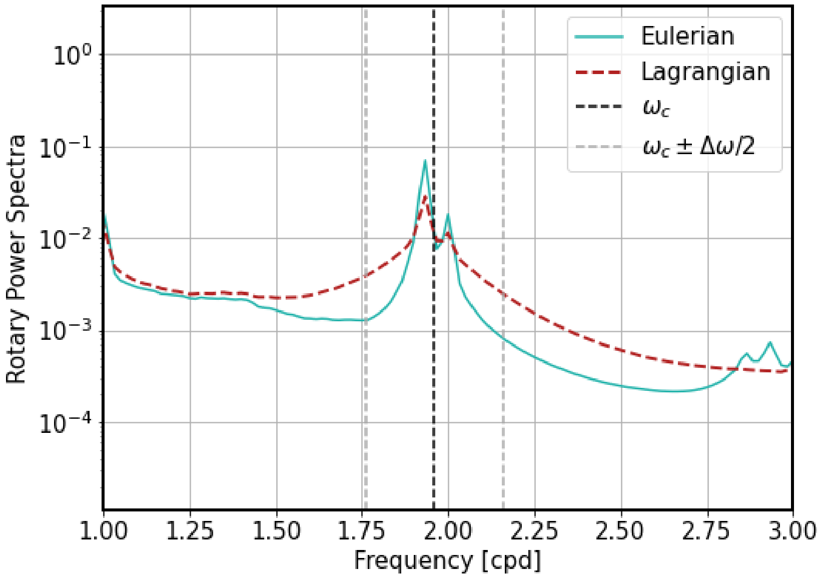

S 2: Semi-diurnal energy peaks of space-averaged power spectra computed from the LLC4320 outputs and offline-simulated drifters.(blue curve) Eulerian and (red dashed curve) Lagrangian semi-diurnal energy peaks. Spectra are obtained by computing the power spectra of the segmented complex horizontal velocity time series, u+iv. The methods used for the segmentation and averaging are the same that the ones described in the methods section. Black dashed line represents the central frequency used in the filter presented in the study, and the grey dashed line the limits of the bandwidth.

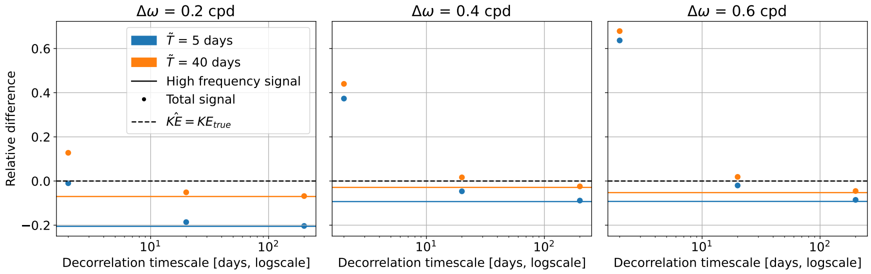

S 3: Difference between true semidiurnal energy and energy estimated from bandpass filtered velocity for synthetic experiments. Each synthetic signal is composed by semidiurnal signal and low-frequency signal. Several set of 500 time series are generated varying internal tides incoherence timescale, $\tilde{T}$=[5,40] days (blue and orange line and points, respectively) and low-frequency motion decorrelation timescale, $\bar{T}$ = [2,20,200] days (x-axis). The bandwidth, $\Delta\omega$, used for filtering also varies: 0.2,0.4,0.6 cpd (from left to right). The black dashed line correspond to the case where true and estimated semidiurnal energy are equal. Colored lines correspond to the semidiurnal energy estimated in the absence of low-frequency motion. They quantify the impact of combined filter and incoherence timescale. Colored points correspond to energy estimates when low-frequency motion is present in the signal before filtering. They quantify the additional energy found in the semidiurnal band due to low-frequency energy, this quantity increases when $\bar{T}$ decreases.
